# Chromosome-scale genomes of commercial timber trees (*Ochroma pyramidale, Mesua ferrea*, and *Tectona grandis*)

**DOI:** 10.1038/s41597-023-02420-8

**Published:** 2023-08-03

**Authors:** Sunil Kumar Sahu, Min Liu, Yewen Chen, Jinshan Gui, Dongming Fang, Xiaoli Chen, Ting Yang, Chengzhong He, Le Cheng, Jinlong Yang, Durgesh Nandini Sahu, Linzhou Li, Hongli Wang, Weixue Mu, Jinpu Wei, Jie Liu, Yuxian Zhao, Shouzhou Zhang, Michael Lisby, Xin Liu, Xun Xu, Laigeng Li, Sibo Wang, Huan Liu

**Affiliations:** 1 State Key Laboratory of Agricultural Genomics, Key Laboratory of Genomics, Ministry of Agriculture, BGI Research, Shenzhen, 518083 China; 2 BGI Life Science Joint Research Center, Northeast Forestry University, Harbin, 150400 China; 3 State Key Laboratory of Subtropical Silviculture, Zhejiang A&F University, 311300 Hangzhou, China; 4 Southwest Forestry University, Kunming, Yunnan 650224 China; 5 BGI Research, Kunming, Yunnan 650106 China; 6 College of Forensic Science, Xi’an Jiaotong University, Xi’an, China; 7 Forestry Bureau of Ruili, Yunnan Dehong, Ruili, 678600 China; 8 Chinese Academy of Forestry, Beijing, China; 9 Laboratory of Southern Subtropical Plant Diversity, Fairy Lake Botanical Garden, Shenzhen, Chinese Academy of Sciences, Shenzhen, 518004 China; 10 Department of Biology, University of Copenhagen, Copenhagen, Denmark; 11 Guangdong Provincial Key Laboratory of Genome Read and Write, BGI Research, Shenzhen, 518083 China; 12 National Key Laboratory of Plant Molecular Genetics and CAS Center for Excellence in Molecular Plant Sciences, Institute of Plant Physiology and Ecology, Chinese Academy of Sciences, Shanghai, 200032 China

**Keywords:** Next-generation sequencing, Comparative genomics

## Abstract

Wood is the most important natural and endlessly renewable source of energy. Despite the ecological and economic importance of wood, many aspects of its formation have not yet been investigated. We performed chromosome-scale genome assemblies of three timber trees (*Ochroma pyramidale, Mesua ferrea*, and *Tectona grandis*) which exhibit different wood properties such as wood density, hardness, growth rate, and fiber cell wall thickness. The combination of 10X, stLFR, Hi-Fi sequencing and HiC data led us to assemble high-quality genomes evident by scaffold N50 length of 55.97 Mb (*O. pyramidale*), 22.37 Mb (*M. ferrea*) and 14.55 Mb (*T. grandis*) with >97% BUSCO completeness of the assemblies. A total of 35774, 24027, and 44813 protein-coding genes were identified in *M. ferrea*, *T. grandis* and *O. pyramidale*, respectively. The data generated in this study is anticipated to serve as a valuable genetic resource and will promote comparative genomic analyses, and it is of practical importance in gaining a further understanding of the wood properties in non-model woody species.

## Background & Summary

Wood being the most important natural and permanently sustainable energy source, plays an important role as an eco-efficient alternative to fossil fuels^1,2^. Wood, also known as secondary xylem, is the major structure that gives stability to woody plants and supplies all other plant tissues with water from the roots. Over the last decades, our knowledge of cellular wood formation (xylogenesis) has increased significantly^2–5^. Wood is formed due to the action of the vascular cambium, which is composed of meristematic initials that generate phloem or xylem precursor cells. Hormonal signals predominate in the proliferation of the cells of the vascular cambium, and the plant hormone auxin plays a critical role in wood formation^6–8^. Although many genes encoding the components (cellulose, xylan, glucomannan, and lignin) of wood biosynthesis have been identified and functionally characterized in poplar and *Arabidopsis*^8–11^, how and which genes particularly affect the wood properties are largely unknown. To tailor wood for our use, it is critical to dissect the molecular and biochemical mechanisms controlling wood formation^12^. The knowledge gained from such studies can be applied to genetically modify the wood quantity and quality^13^.

**Fig. 1:**
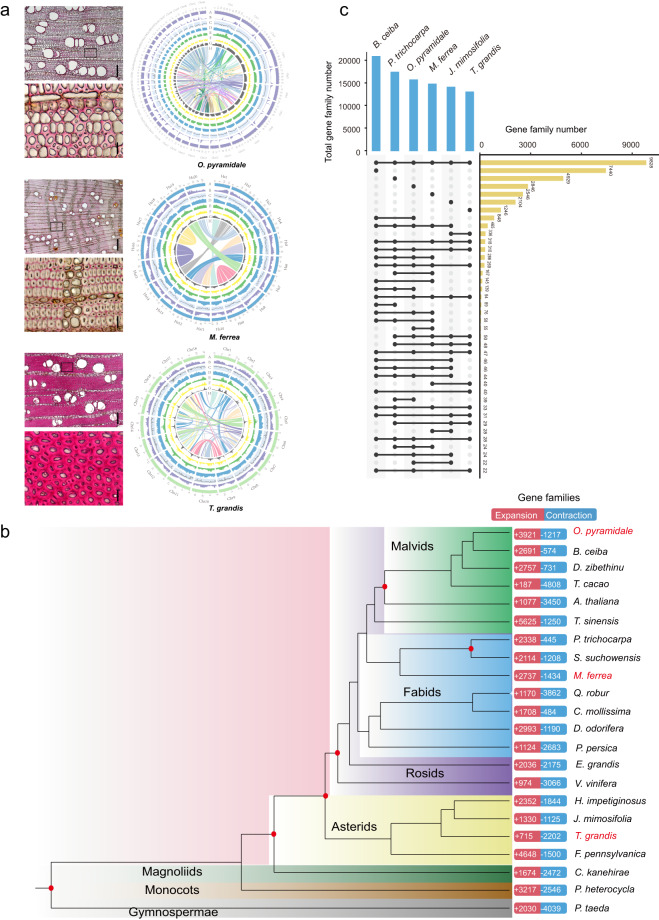
Circos plot, gene family expansion/contraction of three newly sequenced non-model tree genomes. (**a**) Cross-sections of developing tissue stained with phloroglucinol–HCl (the left panel). Bar1 = 200 μm, bar2 = 20 μm stroke. Concentric circles from outermost to innermost (right panel), show (**a**) chromosomes and megabase values, (**b**) gene density, (**c**) GC content, (**d**) repeat density, (**e**) LTR density, (**f**) LTR *Copia* density, (**g**) LTR *Gypsy* density and (**h**) inter-chromosomal synteny (A-H were calculated in non-overlapping 200Kb – 1000Kb sliding windows). (**b**) Phylogenetic tree of 22 species based on low-copy nuclear genes. All nodes exhibit 100% bootstrap support based on maximum likelihood analysis. All the species sequenced in the present study are highlighted in red color. The bar on the right panel shows the number of gene families that are expanded or contracted (p-value ≤ 0.05). (**c**) Comparative analysis of gene family numbers among representative tree species using an upset plot, while the right panel displays the gene family numbers.

With the sequencing of the genomes of increasing numbers of tree species (excluding model plants)^14–20^, it is now possible to uncover the molecular mechanisms controlling the formation of wood. So far, the genome sequences of several important tree species have been released like *Populus trichocarpa*^21^, *Eucalyptus grandis*^22^, *Picea abies* (Norway spruce)^23^, *Broussonetia papyrifera* (Paper Mulberry)^24^, *Morus notabilis* (Mulberry tree)^25^, *Tectona grandis* (Teak tree)^17^, *Dalbergia odorifera*^26^, *Dipterocarpus turbinatus* and *Hopea hainanensis*^27^. Despite the ecological and economic importance of wood, not all aspects of its formation have been unveiled. Therefore, for the present study, we selected three non-model tree species which exhibit different wood properties such as wood density, hardness, growth rate, and fiber cell wall thickness (Fig. 1a, Table S1).

*Ochroma pyramidale* or balsa is a very fast-growing evergreen tree of Malvaceae family that can reach 25 m in 5 years, and prefer to be grown in a warm and humid environment. It is grown for the production of balsa wood, a very soft and light wood, with a coarse, open grain, and it is one of the lightest wood in trade (USDA, 2019) (Table S1). The density of dry balsa wood ranges from 40–340 kg/m^3^, with a typical density of about 160 kg/m^3^. This spongy texture of the wood is due to its large cells that are filled with water (Fig. 1a, Fig. S1).

*Mesua ferrea*, the ironwood, Indian rose chestnut, or cobra’s saffron, is an evergreen species of Calophyllaceae (Table S1). This slow-growing tree is named after the heaviness (high wood density) and hardness of its timber (Fig. 1a), and it typically grows in tropical and sub-tropical regions. The density is 940 to 1,195 kg/m^3^ (59 to 75 lb/ft3) at 15% moisture content. Since it is difficult to saw, it is mostly used for railroad ties and heavy structural timber (FAO, 2016) (Fig. S2).

Teak (*Tectona grandis L.f*.;2n = 2x = 36) is a tropical and deciduous hardwood tree species belonging to Lamiaceae family (Table S1). It can survive and grow in a variety of climatic and edaphic conditions, but it thrives in a warm, moist, tropical climate with a wide difference in dry and wet seasons. Teak is a highly prestigious wood because of its look, strength (intermediate wood density, Fig. 1a) and resistance to decay and is commonly used in the construction of ships, boats, furniture and aesthetic needs^28^, including several medicinal properties^29^ (Fig. S3).

**Fig. 2:**
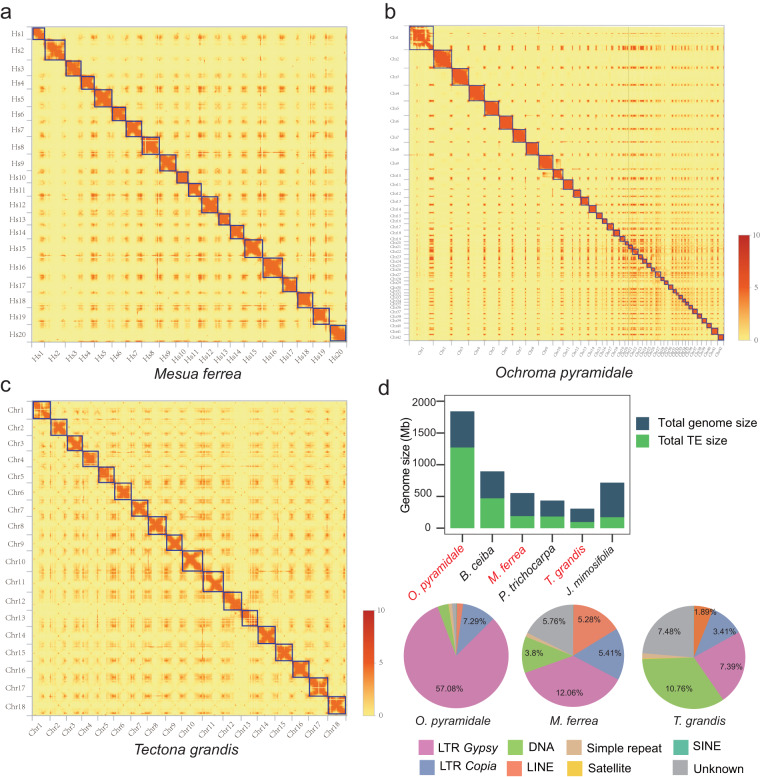
The Hi-C map and the distribution of transposable elements (TEs) across different timber species. Panels (**a**–**c**) present the genome-wide all-by-all interactions captured by the Hi-C map. The map showcases the detailed structure of individual chromosomes, which are scaffolded and assembled independently. The heat map exhibits a color gradient ranging from light orange to dark orange, representing the varying frequencies of Hi-C interaction links, with lighter colors indicating lower frequencies (0) and darker colors indicating higher frequencies (10). (**b**) Distribution of TE types among *O. pyramidale*, *M. ferrea* and *T. grandis*.

Considering the lacunae of key genomic resources with respect to wood formation, fast growth, and genetic architecture of woody plants, we present high-quality chromosome-scale genomes of three economically important timber trees which display varying growth and wood traits. A total of 35774, 24027, and 44813 protein-coding genes were identified in *M. ferrea*, *T. grandis* and *O. pyramidale*, respectively. Based on the K-mer analysis, the genome sizes were estimated to be 1,884 Mb, 534 Mb and 296 Mb for *O. pyramidale*, *M. ferrea* and *T. grandis*, respectively (Table S2). The final assembled genomes showed 552.7 Mb and 306.08 Mb with scaffold N50 values of 0.76 Mb and 0.34 Mb for *M. ferrea* and *T. grandis*, respectively (Figs.  1a, 2, Figs. S4, S5, Table 1, Table S3). While for *O. pyramidale*, the preliminary genome assembly based on the sequencing reads generated by Hi-Fi technology reached 1.84 Gb with a contig N50 of 42.7 Mb (Table S3). We discovered the existence of whole genome duplications (WGDs) in all three timber trees. Noticeably, *O. pyramidale* retained huge numbers of WGD genes compared to others, and the post-WGD retained duplicated gene pairs likely triggered the huge expansion and expression of several wood-formation related TFs (NAC and MYBs), auxin hormone signaling, nutrition and energy supply, and CAzyme-related genes in the fast-growing *O. pyramidale* compared to other tree species. The comprehensive data of these forest trees will serve as a valuable genetic resource, and thus it is of practical importance in gaining further understanding of the complex process of wood biosynthesis, and the basis of the physical and chemical properties of wood.

**Table 1:** The assembly and annotation statistics.

	Mesua ferrea^*^	*Tectona grandis*^#^	*Ochroma pyramidale*^$^
**Genome assembly and annotation**			
Estimated genome size (Mb)	533.68	309.00	1,884.25
Assembly size (Mb)	552.73	306.08	1,840.88
GC content (%)	35.27	33.29	35.56
Contig N50 (Kb)	42.25	88.46	42,724.82
Scaffold N50 (Kb)	760.47	5,835.65	—
BUSCO completeness of assembly (%)	97.3	97.5	97.1
Complete single copy (%)	81.9	93.9	60.8
Complete duplicated (%)	15.4	3.6	36.3
Total number of genes	35,774	24,027	44,813
Average gene length (bp)	3,094.78	3,908.98	3,490.19
DNA mapped reads (%)	95.23	99.04	99.97
BUSCO completeness of annotation (%)	93.70	93.30	94.90
**Pseudochromosome level assembly**			
Total length of pseudochromosome assembly (Mb)	426.34	266.74	1,794.08
Pseudochromosome number	20	18	42
Scaffold N50 (Kb)	22,374.77	14,553.30	55,069.68
BUSCO completeness of pseudochromosome assembly (%)	96.9	96.8	97.1
The rate of pseudochromosome anchored genome (%)	77.13	87.15	97.50

*Genome sequenced by 10X sequencing method; ^#^Genome sequenced by Single Tube Long Fragment Read (stLFR) technology; ^**$**^Genome sequenced by PacBio-CCS (HiFi).

## Methods

### Sample collection, DNA/RNA extraction, library construction and sequencing

The fresh young leaves of *M. ferrea* (HCNGB_00001601) and *T. grandis* (HCNGB_00001711) were collected from Ruili Botanical Garden, Yunnan, China, while samples of *O. pyramidale* (HCNGB_ 00009295) were procured from Xishuangbanna Tropical Botanical Garden, Yunnan. The DNA was extracted using CTAB (cetyltrimethylammonium bromide) method^30^. The amount of DNA extracted from each sample was determined using a Qubit 4.0 fluorometer (Invitrogen, USA). The quality of the extracted DNA was assessed using a NanoDrop spectrophotometer (Thermo Fisher Scientific, USA). The DNA was considered pure if the OD260/280 ratio was between 1.8 and 2.0, and the OD260/230 ratio was between 2.0 and 2.2. The molecular weight of the DNA was evaluated using pulse-field gel electrophoresis (PFGE), and the DNA above 40 kilobases (Kb) was considered for library construction. The GEM and barcode sequences were generated based on the standard protocol (Chromium Genome Chip Kit v1, 10X Genomics, Pleasanton, USA) for *M. ferrea* and *O. pyramidale* (Fig. S4). The barcode libraries were sequenced on a BGISEQ-500 platform to generate 150-bp read pairs^31^. For *Tectona grandis* the library construction and sequencing were performed using the Single Tube Long Fragment Read (stLFR) technology^32^, a method that allows data from long DNA molecules to be sequenced using low-cost second-generation sequencing technology. The library was sequenced on a BGISEQ-500 platform to generate 100-bp and 140-bp reads for read1 and read2, respectively (Table S4). We constructed a SMRTbell target size library using 15 µg high molecular weight (HMW) genomic DNA and used the standard methodology for PacBio-HiFi sequencing. We used Sequencing Primer V2 and Sequel II Binding Kit 2.0 in Grandomics to shear genomic DNA to expected size of fragments for sequencing on a PacBio Sequel II instrument (Table S4).

We also collected branch samples from each tree species to collect xylem and phloem tissues^33^. The fresh young leaves, phloem and xylem were used to extract the RNA by using the PureLink RNA Mini Kit (Thermo Fisher Scientific, Carlsbad, CA, USA). The samples with RNA integrity number (RIN) value above seven were considered for further sequencing. RNA libraries were constructed by using the TruSeq RNA Sample Preparation Kit (Illumina, San Diego, CA, USA), and were then sequenced on the BGISEQ-500 platform (paired-end, 100-bp reads or 150-bp reads) (Table S5). The RNA reads were filtered by the Trimmomatic^34^ with the parameters: ILLUMINACLIP:adapter.fa:2:30:20:8:true HEADCROP:5 LEADING:3 TRAILING:3 SLIDINGWINDOW:5:8 MINLEN:50.

### Hi-C library construction and sequencing

The Hi-C libraries were constructed by utilizing the MboI restriction enzyme and following the *in situ* ligation protocols^35^. The chromatin digested with MboI was labeled at the ends with biotin-14-dATP (Thermo Fisher Scientific, Waltham, MA, USA) and employed for *in situ* DNA ligation. Subsequently, the DNA was extracted, purified, and sheared using Covaris S2 (Covaris, Woburn, MA, USA). Following A-tailing, pull-down, and adapter ligation, the DNA libraries were subjected to sequencing on a BGISEQ-500 to generate 100-bp read pairs (Table S6). Hi-C data enabled the identification of 42, 20 and 18 chromosomes for *O. pyramidale*, *M. ferrea* and *T. grandis*, respectively, which was consistent with their reported chromosome numbers (Fig. 1a)^17^.

### Evaluation of genome size

The obtained DNA sequencing reads from the 10X and stLFR libraries were filtered using SOAPnuke^36^ with the parameters (-l 10 -q 0.1 -n 0.01 -Q 2 -d --misMatch 1 --matchRatio 0.4). Clean reads from paired-end libraries were used to estimate genome sizes (Table S2, Table 1). To conduct the k-mer frequency distribution analysis, the following formula was employed:

$$Gen=Num* \left(Len-17+1\right)/K\_Dep$$

Where, *Num* = read number of reads used, *Len* = the read length, *K* = k-mer length, and *K_Dep* = main peak’s location.

### Genome assembly

We used Supernova (version 2.1.1)^37^, a *de novo* assembly program designed to assemble diploid genomes using Linked-Reads (10X, stLFR libraries sequences) using the default parameters, and exported into fasta format using the ‘pseudohap2’ style. The GapCloser^38^ was used to fill the gap using the parameters “-l 150” for each species except *Tectona grandis*. For Hi-fi reads, first, we use CCS (version 6.0.0) with the parameter –min-passes 3, then samtools (version 1.11)^39^ to convert the bam file to the fastq file. The fastq was then used as the input file for hifiasm software^40^ with the default parameters.

The Hi-C reads were quality controlled and aligned to each species’ genome assembly using Juicer with default settings^41^. Next by using the 3D-DNA pipeline (with default parameters)^42^, an initial assembly at the superscaffold-level was automatically generated. This corrected mis-joins, arranged the scaffolds in the proper order and orientation, and organized them from the initial draft assembly. Manual inspections and refinements of the draft assembly were performed using Juicebox Assembly Tool^43^ to ensure accuracy. From the Hi-C interaction map in Fig. 2, it appears that different chromosomes interact repeatedly (Fig. S5). This phenomenon is quite common, and has been observed in other organisms as well, such as the cotton genome^44^, Kiwifruit^45^, and Phoebe tree^46^. There are a number of possible explanations for this phenomenon. One possibility is that it is due to the presence of repetitive DNA sequences in the genome. These sequences are often found in tandem repeats, which means that they are repeated multiple times in close proximity to each other. This can lead to increased levels of interaction between different chromosomes, as the repetitive sequences can bind to each other. Another possibility is that the repeated interaction is due to the presence of functional elements that are shared between different chromosomes. For example, there are a number of genes that are involved in DNA repair and replication, and these genes are often found on multiple chromosomes. This means that the chromosomes that contain these genes may interact with each other more frequently, as they are likely to be involved in the same biological processes.

### Repeat annotation

To characterize the repeat elements based on homology, the alignment of the genome assembly was performed using Repbase v.21.01^47^ and RepeatMasker v.4.0.6^48^. For the *de novo* approach, RepeatModeler v.1.0.8^49^ and LTR Finder v.1.0.6^50^ were employed to construct a *de novo* repeat library using the genome assembly. Subsequently, RepeatMasker v.4.0.6^48^ was used to identify and annotate repeat elements in the genome. Tandem repeats in the genome were annotated using TRF v. 4.07^51^. Finally, the repetitive regions of the genome were masked prior to gene prediction.

Both *M. ferrea* and *T. grandis* displayed comparatively low repetitive elements (REs), which accounted for 33.93% (188.11 Mb) and 31.3% (95.80 Mb) of assemblies, respectively (Fig. 2d, Table S7). While *O. pyramidale* contains 1269.55 Mb sequences representing 68.96% of the assembled genome, particularly long terminal repeat (LTR) *Gypsy* and *Copia* types of retrotransposons which accounted for ~64% of total genome size.

### Gene annotation analysis

The gene structures were predicted using the MAKER-P pipeline (version 2.31)^52^, which relied on RNA, homologous, and *de novo* prediction evidence (Table 1). To obtain the RNA evidence, the clean transcriptome reads were assembled into inchworms using Trinity (version 2.0.6)^53^, and these sequences were subsequently supplied to MAKER-P as expressed sequence tags.

To perform homologous comparisons, we obtained protein sequences from either the model plant or closely related species for each of the species being analyzed. For *de novo* prediction, we created several training sets to optimize various *ab initio* gene predictors. Initially, we employed a genome-guided approach using Trinity^53^ to generate a set of transcripts. These transcripts were then mapped back to the genome using PASA (version 2.0.2)^54^, resulting in the generation of gene models that possessed realistic gene characteristics such as size, number of exons/introns per gene, and splicing site features. The complete gene models selected in Augustus^55^ were used for training. Genemark-ES (version 4.21)^56^ was self-trained using default parameters.

The initial phase of MAKER-P utilized the aforementioned evidence, employing default parameters except for “est2genome” and “protein2genome,” which were set to “1.” This resulted in the generation of gene models supported solely by RNA and protein data. Subsequently, SNAP^57^ was trained using these gene models. The second and final rounds of MAKER-P were executed with default parameters, culminating in the production of the final gene models.

### Functional annotation

Protein-coding genes were functionally annotated by aligning the predicted amino acid sequences with public databases, using sequence similarity and domain conservation as criteria (Table 1). To identify the best matches, the annotation process involved searching the protein-coding genes against protein sequence databases, including the Kyoto Encyclopedia of Genes and Genomes (KEGG)^58^, the National Center for Biotechnology Information (NCBI) non-redundant (NR) and KOG databases^59^, SwissProt^60^ and TrEMBL using BLASTP with an E-value cut-off of 1e-5. Then, InterProScan 55.0 (InterProScan)^61^ was used to identify domains and motifs based on Pfam^62^, SMART^63^, PANTHER^64^, PRINTS^65^ and ProDom^66^ (Table S8).

### Annotation of non-coding RNAs

Ribosomal RNA (rRNA) genes were searched against the *A. thaliana* rRNA database using BLAST. MicroRNAs (miRNA) and small nuclear RNA (snRNA) were searched against the Rfam database^62^. tRNAscan-SE was also used to scan for tRNAs^67^ (Table S9).

### Identification of gene families and phylogenetic analysis

The protein-coding genes of 22 representative species were selected for gene families analysis using OrthoFinder (version 2.3.14)^68^ with default parameters (Fig. 1b, Table S10). However, we only obtained 5 single-copy orthogroups, which was not enough to construct a phylogenetic tree. Therefore, we added some low-copy orthogroups (which contain more than one gene per species) to improve the robustness of our tree. Totally, 364 low-copy orthogroups were used for phylogenetic tree construction. The protein sequences from the 364 low-copy orthogroups were extracted and aligned by using MAFFT (version 7.310)^69^. We used a Perl script to trim the gaps of the aligned sequences, and then concatenated them into a supergene sequence. The phylogenetic tree was subsequently constructed by IQ-TREE (version 1.6.1)^70^ with “-bb 2000 -alrt 1000”. *Tectona grandis* genomes were compared with the previously published genome using nucmer (version 4.0.0rc1) (Fig. S6). The results showed that stLFR was able to generate high-quality sequence data that was comparable to PacBio sequencing (Table S11). By offering a more cost-effective and computationally efficient alternative to PacBio sequencing, stLFR could make genomic data more accessible to a wider range of researchers.

Next, we identified that most gene families of *O. pyramidale*, *M. ferrea* and *T. grandis* were commonly shared including other representative trees, but 2846, 2546 and 1246 gene families appeared to be unique in genomes of *O. pyramidale*, *M. ferrea* and *T. grandis*, respectively (Fig. 1c). The homolog matrix of orthogroups was analyzed to infer ancestral and lineage-specific gene family dynamics along the phylogenetic tree, resulting in the expansion of 3921, 2737 and 715 gene families, while 1217, 1434 and 2202 were contracted in genomes of *O. pyramidale*, *M. ferrea* and *T. grandis*, respectively. The number of expanded gene families in *O. pyramidale* and *M. ferrea* was remarkably higher than *T. grandis* (Fig. 1b).

### Whole-genome duplication (WGD) and single-gene duplication

The DupGen_finder (version 5.16.3)^71^ was used to identify different modes of duplicated gene pairs, such as WGD, tandem duplication, proximal duplication, transposed duplication, dispersed duplication, and the number of gene pairs are shown in Table S12. The coding sequences were used for estimating a synonymous substitution rate (Ks) using the wgd software (version 1.0)^72^. Then, we constructed the Ks distribution plot using the Ks values of the WGD gene pairs.

**Fig. 3:**
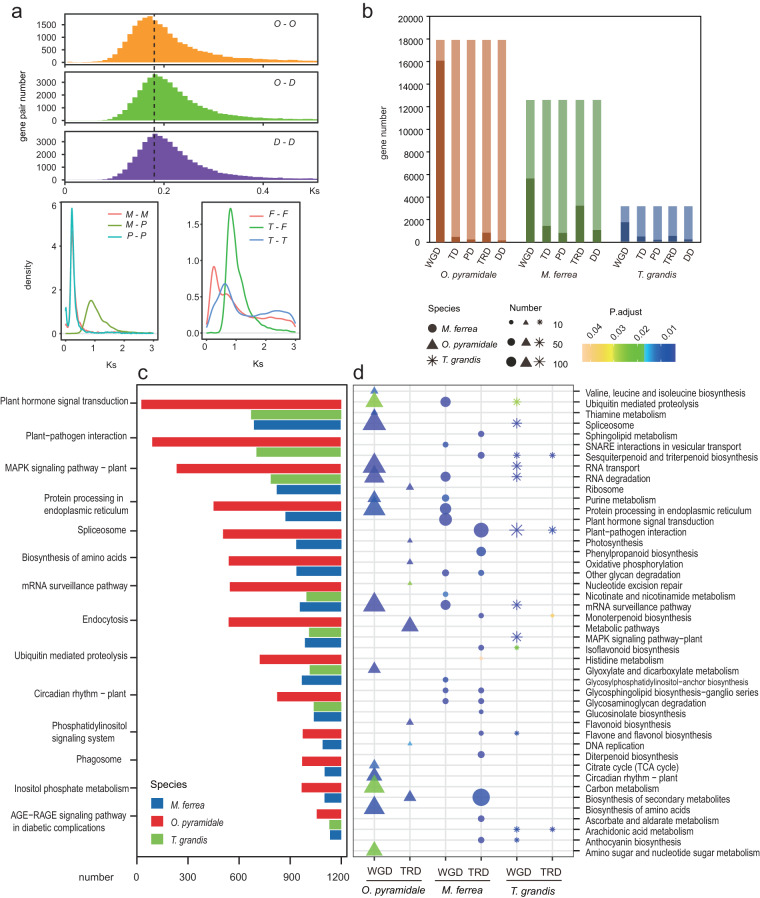
Genome evolution and WGD analysis of timber species. (**a**) The distribution of Ks. Here, *O-O* means the paralogous of *O. pyramidale*, O-D means the orthologs of *O. pyramidale* and *D. zibethinu, D-D* means the paralogous of *D. zibethinu*, *M-M* means the paralogous of *M. ferrea, M-P* means the orthologs of the *M. ferrea* and *P*. *trichocarpa*, *P-P* means the paralogous of *P*. *trichocarpa, F-F* means the paralogous of *F. pennsylvanica*, *T-F* meads the orthologs of *T. grandis* and *F. pennsylvanica*, and *T-T* means the paralogous of *T. grandis*. (**b**) Bar chart showing the number of gene duplication in various duplicated modes and gene duplication-induced expanded gene number. Light color: Total gene number in expanded gene families in each species, while dark color represents specific duplication types (WGD: WGD duplicate; TD: tandem duplication; PD: proximal duplication; TRD: Transposed duplications. (**c**) The total number of KEGG enriched WGD genes among the representative timber species *Ochroma pyramidale, Mesua ferrea*, *Tectona grandis*. (**d**) The KEGG enrichment of WGD and TRD duplication-induced expansion in gene families in each species.

Whole genome duplication (WGD) is considered to be the main factor driving genome evolution and expansion^73–75^. The distribution of Ks for the orthologs of *O. pyramidale* and *Durio zibethinu* showed peak at ~0.19 (Fig. 3a). While their paralog peaks at 0.15 and 0.17 for *O. pyramidale* and *D. zibethinu* suggested that they experienced one lineage-specific WGD after the divergence. *T. grandis* and *Fraxinus pennsylvanica*^76^ displayed individual WGD events after their divergence (Fig. 3a).

Notably, we observed a large number of paralogous genes retained in *O. pyramidale* genome after its specific WGD event compared to other selected species (Fig. 3b,c). KEGG enrichment analysis indicated the WGD duplicated gene pairs in *O. pyramidale* retained several functions such as plant hormone signal transduction, MAPK signaling pathway, and circadian rhythm (Fig. 3c, Table S13). Next, we investigated the WGD duplication-induced expansion in gene families by combining and overlapping WGD duplicated gene pairs and expanded gene families (EGFs). We identified a total of 42,855, 32,804 and 208,22 duplicated genes from *O. pyramidale, M. ferrea* and *T. grandis* genomes, respectively, which were classified into five categories, that is, the WGD duplicates, tandem duplicates (TD), transposed duplicates (TRD), proximal duplicates (PD) and dispersed duplicates (DD) (Table S14). Interestingly, WGD events contributed to the highest proportion of expansion of the gene families compared with other duplication types in *O. pyramidale* (89.74%) (Fig. 3b). However, *O. pyramidale* contains abundant TEs, a relatively low proportion of expanded gene families caused by TRD compared to *M. ferrea*. For *M. ferrea*, a higher proportion of expanded gene families was induced by TRD and TD but not WGD (Fig. 3a).

KEGG enrichment of expanded gene families induced by WGD indicated that genes related to Plant hormone signal transduction, Photosynthesis, MAPK signaling pathway, Nitrogen metabolism, alpha−Linolenic acid metabolism, Biosynthesis of unsaturated fatty acids, Fatty acid metabolism etc., might be mainly caused by WGD events in *O. pyramidale* (Tables S13, S4, Fig. 3d).

### Identification of transcription factors (TFs), copy number normalization and gene expression analyses

The protein sequences were downloaded from the Plant Transcription Factor Database (PlantTFDB)^77^, and aligned by MAFFT (version 7.310)^69^, later used for HMMs (Hidden Markov Models) construction for each type of TFs by using Hmmer (version3.1b2)^78^.

The protein sequences of the representative species were searched against the HMMs of different type TFs by HMMsearch (version 3.1b2)^78^, and filtered with an e-value cutoff of 1e-5. Subsequently, the protein sequences of ARF, HD-Zip, WOX, MYB, and NAC gene families of each species were aligned by MAFFT^69^, and the gene family trees were constructed by IQ-TREE (version 1.6.1)^70^ with the parameters “-bb 1000”. Instead of directly comparing gene copy numbers among selected species, we normalized the data based on the genome size, and then performed the comparison, avoiding any sort of data bias^79^.

**Fig. 4:**
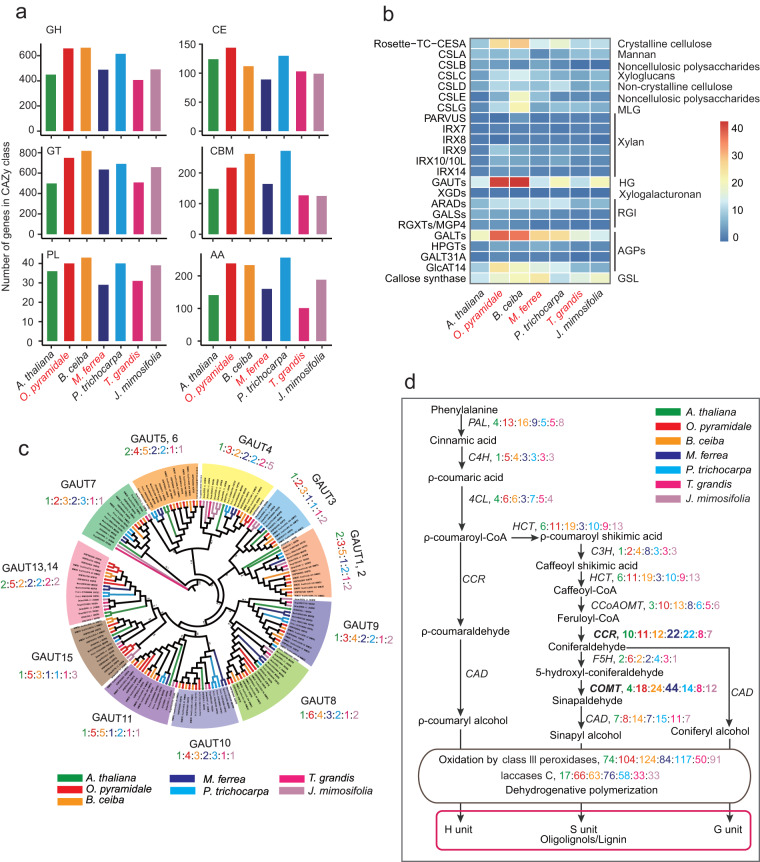
Comparative analyses of gene copy numbers of cell wall-related genes. (**a**) The number of genes in the GH, CE, GT, CBM, PL, and AA family of the Carbohydrate Active Enzymes database (CAZy) (http://www.cazy.org/) in seven species. GH: Glycoside hydrolase family, CE: Carbohydrate esterase family, GT: Glycosyltransferase family, CBM: Carbohydrate-binding module family, PL: Polysaccharide lyase family, AA: Auxiliary Activities family. (**b**) The heatmap of gene copy numbers of cell wall biosynthesis-related genes. (**c**) The phylogenetic tree (IQ) of GAUT gene family with 5000 bootstrap replicates. Different colors represent different species. (**d**) The copy numbers of the respective genes in monolignol synthesis pathway. Phenylalanine ammonia lyase (PAL), cinnamate 4-hydroxylase (C4H), 4-coumarate coenzyme A ligase (4CL), Ferulate 5-hydroxylase (F5H), p-coumarate 3-hydroxylase (C3H), p-hydroxycinnamoyl-CoA:quinate/shikimate hydroxycinnamoyl transferase (HCT), caffeoyl-CoA O-methyltransferase (CCoAOMT), cinnamoyl-CoA reductase (CCR), caffeate/5-hydroxyferulate 3-O-methyltransferase (COMT), and cinnamyl alcohol dehydrogenase (CAD), Laccases (LAC).

Plant rapid growth needs extensive remodeling and modifications of cell wall to allow the cell wall to be flexible^80–82^; we thus investigated the enzymes of carbohydrate metabolism, including the glycoside hydrolases (GH) and glycosyltransferases (GT)^80^. We selected the protein sequences which are involved in the synthesis and regulation of the cell wall components, auxins, ethylene and salicylic acid in *Arabidopsis* as query, and blast with the protein sequences of representative species with an e-value cutoff of 1e-5, and then checked the gene function by SwissProt annotation. The two Malvaceae genomes contained 658/664 GH and 748/818 GT genes in *O. pyramidale* and *B. ceiba*, respectively, which is remarkably higher than other selected species (Fig. 4a, Table S15). Next, we investigated GTs related to the cell wall assembly. The copy number of the genes involved in cellulose synthesis of the primary cell wall in *O. pyramidale* was higher than *M. ferrea*. Also, the copy numbers of genes involved in hemicellulose synthesis of cell wall in *O. pyramidale* were higher than *M. ferrea* and other tree species (Fig. 4b, Table S16). Additionally, the copy number of GAUTs (galacturonosyltransferases) were more than two-fold higher than other selected species. GAUTs are mainly involved in pectin and/or xylan biosynthesis in cell walls, thereby affecting the overall growth of the tree and wood plasticity^83^. Furthermore, our phylogenetic analysis using the IQ tree grouped GAUTs into eleven sub-groups, and GAUT11/8/15 and 5,6 particularly showed higher expansion in *O. pyramidale* compared to other species (Fig. 4c, Table S17).

Lignin is one of the main components of trees or plants, constituting around 30% of the dry mass of wood, and lignin gives trees their rigidity^12,84,85^. To explore the likely reason behind the heaviness (high wood density) of *M. ferrea*, we compared the genes involved in the lignin biosynthesis in *O. pyramidale* and other tree species. We found Caffeic acid O-methyltransferases (COMT) and Cinnamoyl CoA Reductase (CCR) exhibited extensive expansion in *M. ferrea* (Fig. 4d, Table S18).

## Data Records

The raw sequence data reported in this paper have been deposited in the Genome Sequence Archive^86^ in the National Genomics Data Center^87^, China National Center for Bioinformation/Beijing Institute of Genomics, Chinese Academy of Sciences (GSA: CRA011294)^88^ that are publicly accessible. The assembled contigs or scaffolds genomes are submitted to the Genome Warehouse under the accession number GWHDOCN00000000^89^, GWHDOCP00000000^90^ and GWHDOCQ00000000^91^ of *O. pyramidale*, *M. ferrea* and *T. grandis*. The assembled pseudo-chromosome genomes were submitted to the Genome Warehouse under the accession number GWHDOCO00000000^92^, GWHDOCR00000000^93^ and GWHDOCS00000000^94^ of *O. pyramidale*, *M. ferrea* and *T. grandis*. The data sets generated and analyzed during the current study are also available in the CNGB Nucleotide Sequence Archive (CNSA) under accession numbers CNP0001860 and CNP0001861.

The annotation files are available in Figshare^95^. All other data generated or analyzed during this study are included in this article and its supplementary information files. WGS data for *Tectona grandis*^96^, and *Mesua ferrea*^97^ from PRJNA438407 were obtained from the respective accession numbers for the genome size estimation only.

## Technical Validation

Completeness assessment was performed using BUSCO (Bench-marking Universal Single-Copy Orthologs) version 3.0.1^98^ with Embryophyta odb9 database. From the 1,375 core Embryophyta genes, 1,338 (97.3%), 1,329 (96.6%), 1,340 (97.5%), 1,323 (96.2%), 1,340 (97.5%), 1,335 (97.0%) and 1,335 (97.1%) were identified in the *M. ferrea, D. sissoo, K. senegalensis, S. macrophylla*, *T. grandis* and *O. pyramidale*, respectively (Supplemental Tables 19, 20). To further evaluate the completeness of the assembled genome, we performed short reads mapping using clean raw data. In total, 95.23%, 98.54%, 99.04%, 97.68%, 97.43%, and 98.05% reads were mapped to the genomes, among which 83.56%, 87.63%, 94.39%, 82.72%, 90.55%, and 90.61% of them were properly paired to *M. ferrea, D. sissoo, K. senegalensis, S. macrophylla*, *T. grandis* and *O. pyramidale*, respectively. The transcriptome sequences were assembled by using Bridger tool^99^, then mapping to the scaffold assembly was performed by using the BLAT software^100^ (Table S5). The BUSCO analysis was again performed after the Hi-C assembly which gave similar results as that of 10X and stLFR genome assemblies (Supplemental Tables 21, 22).

### Supplementary information

Supplementary Table and Figure captions

Supplementary Figures

Supplementary Tables

## Data Availability

The codes and pipelines used in data processing were all executed according to the manual and protocols of the corresponding bioinformatics software. The specific versions of software have been described in Methods. However, the following perl script was used to trim the gaps of the aligned sequences. #! usr/bin/perl -w use strict; sub usage{ print STDERR «USAGE; usage: $0 <phy.file> <cut off> USAGE USAGE exit; } my $file = shift; open IN,”$file” or die $!; open OUT,” >./$file.trim.phy” or die $!; my ($num_species,$len); my (@name,@seq,@gap_ratio,@match_ratio); my %new_seq; my @u_site; my $i = 0; my $resultfile = “./$file.trim.phy”; while (<IN>){ chomp; if (/^\s+(\d+)\s+(\d+)/){ $num_species = $1; $len = $2; next; } my @temp = split; $name[$i] = $temp[0]; @{$seq[$i]} = (split //,$temp[1]); $i++; } print “$num_species\n”; close IN; for (my $j = 0;$j <$len;$j++){ my ($gap,$match) = (0,0); for (my $i = 0;$i < $num_species;$i++){ if ($seq[$i][$j] eq “-“){ $gap++; }else { $match++; } } $gap_ratio[$j] = $gap/$num_species*100; $match_ratio[$j] = $match/$num_species*100; } for (my $i = 0;$i < $num_species;$i++){ for (my $j = 0;$j < $len;$j++){ if ($gap_ratio[$j] >  = $ARGV[0]){ next; }else { $new_seq{$i}. = $seq[$i][$j]; } } print OUT “$name[$i]\t$new_seq{$i}\n”; } my $len_temp = length($new_seq{0}); print “$len_temp\n”; ‘sed -i ‘1i $num_species $len_temp’ $resultfile‘;
