# Supplementary material for: Chromosome-scale genomes of commercial timber trees (Ochroma pyramidale, Mesua ferrea, and Tectona grandis)
Source: Sci Data. 2023 Aug 3;10:512. doi: 10.1038/s41597-023-02420-8 (PMC10400565; doi:10.1038/s41597-023-02420-8)
Supplement: Supplementary file 1 — Supplementary Table and Figure captions [file 41597_2023_2420_MOESM1_ESM.docx]

**Description of Additional Supplementary Files**

Table S1. Description of physical traits of the three sequenced timber trees.

Table S2. Use of WGS data for genome size estimation.

Table S3. The genome assembly results of *Mesua ferrea*, *Tectona grandis*, and *Ochroma pyramidale*.

Table S4. DNA Sequencing statistics of three species.

Table S5. RNA sequencing statistics of three species.

Table S6. Hi-C evaluation.

TableS7. The repeat element annotation of *Mesua ferrea*, *Tectona grandis*, *Ochroma pyramidale*.

Table S8. The functional annotation.

TableS9. The Annotation of non-coding RNA genes in the genomes of *Mesua ferrea*, *Tectona grandis*, *Ochroma pyramidale*.

Table S10. The list of species used for phylogenetic tree construction and download source.

Table S11. Comparison of the assembly and annotation statistics between Tectona grandis and the previously published *Tectona grandis*.

Table S12a. The number of genes of different duplication type between each species.

Table S12b. The numbers of genes which expanded after duplication events.

Table S13. The KEGG and GO enrichment of the expanded gene families.

Table S14. the KEGG enrichment of different duplication event genes which expanded in each species.

Table S15. The gene numbers in CAZy database.

Fig. S1 Morphological features of *Ochroma pyramidale*.

Fig. S2 Morphological features of *Mesua ferrea*.

Fig. S3 Morphological features of *Tectona grandis*.

Fig. S4 Sampling sites and genome analysis workflow.

Fig. S5 The Hi-C map of *Ochroma pyramidale*.

Fig. S6 Sequence alignment dot plots generated with NUCmer (version 4.0.0rc1) comparing the chromosome sequences of *Tectona grandis* from this study to the previously published version.
