# Supplementary material for: Chromosome-scale genomes of commercial timber trees (Ochroma pyramidale, Mesua ferrea, and Tectona grandis)
Source: Sci Data. 2023 Aug 3;10:512. doi: 10.1038/s41597-023-02420-8 (PMC10400565; doi:10.1038/s41597-023-02420-8)
Supplement: Supplementary file 2 — Supplementary Figures [file 41597_2023_2420_MOESM2_ESM.pdf]

Supplemental Information

Chromosome-scale genomes of commercial timber trees (*Ochroma pyramidale, Mesua ferrea*, and *Tectona grandis*)

Sahu et al

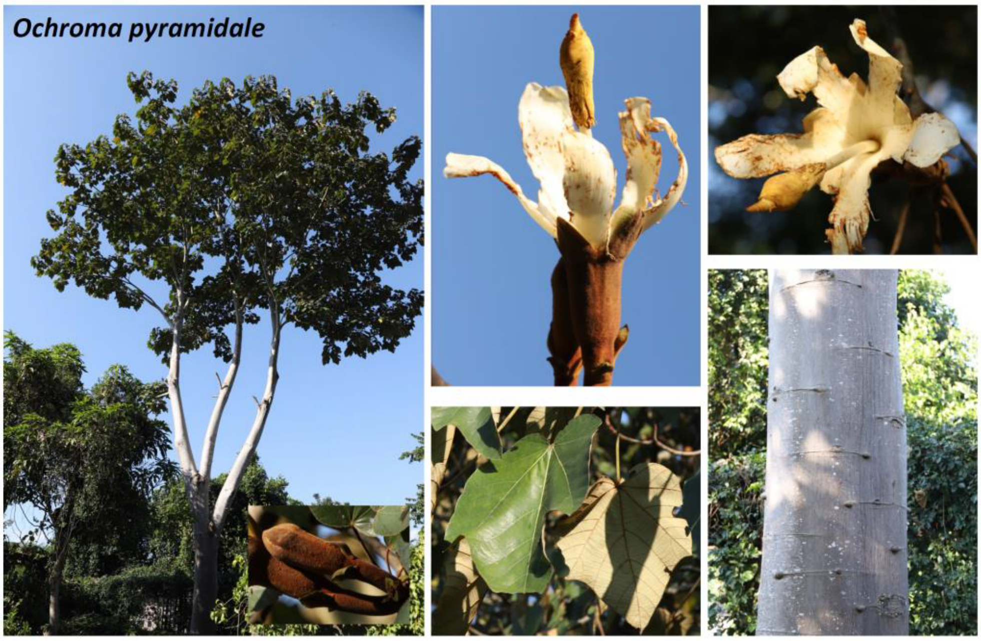

Fig. S1 Morphological features of *Ochroma pyramidale.*

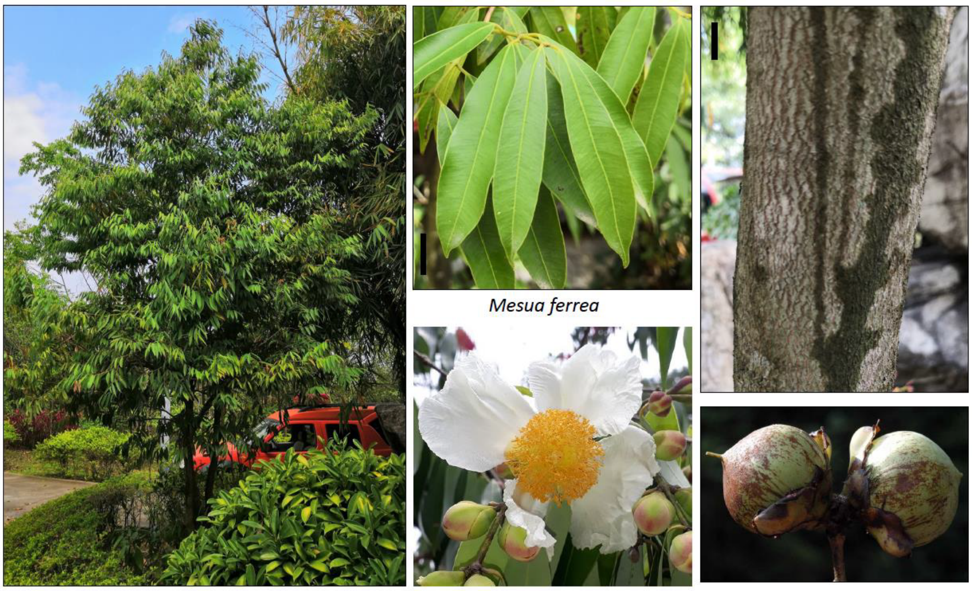

Fig. S2 Morphological features of *Mesua ferrea.*
Photos of flower and seeds are obtained from
https://www.inaturalist.org/observations/24351299
https://indiabiodiversity.org/biodiv/img//Mesua_ferrea/mesuferr_15.jpg

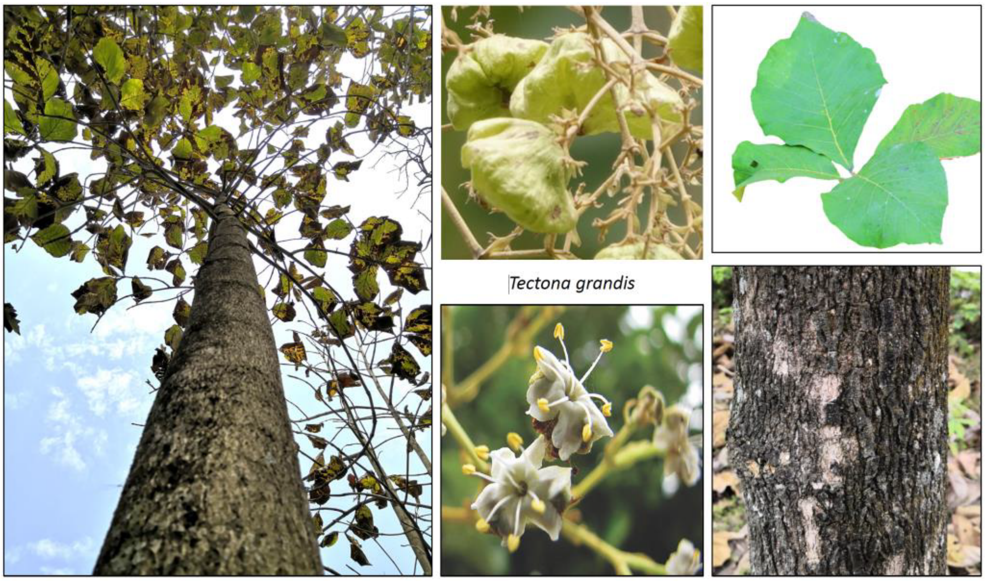

Fig. S3 Morphological features of *Tectona grandis.*
Photos of flower and fruit are obtained from https://indiabiodiversity.org/biodiv/img//Tectona%20grandis/Tectona_grandis_flower.jpg https://www.inaturalist.org/observations/67507739

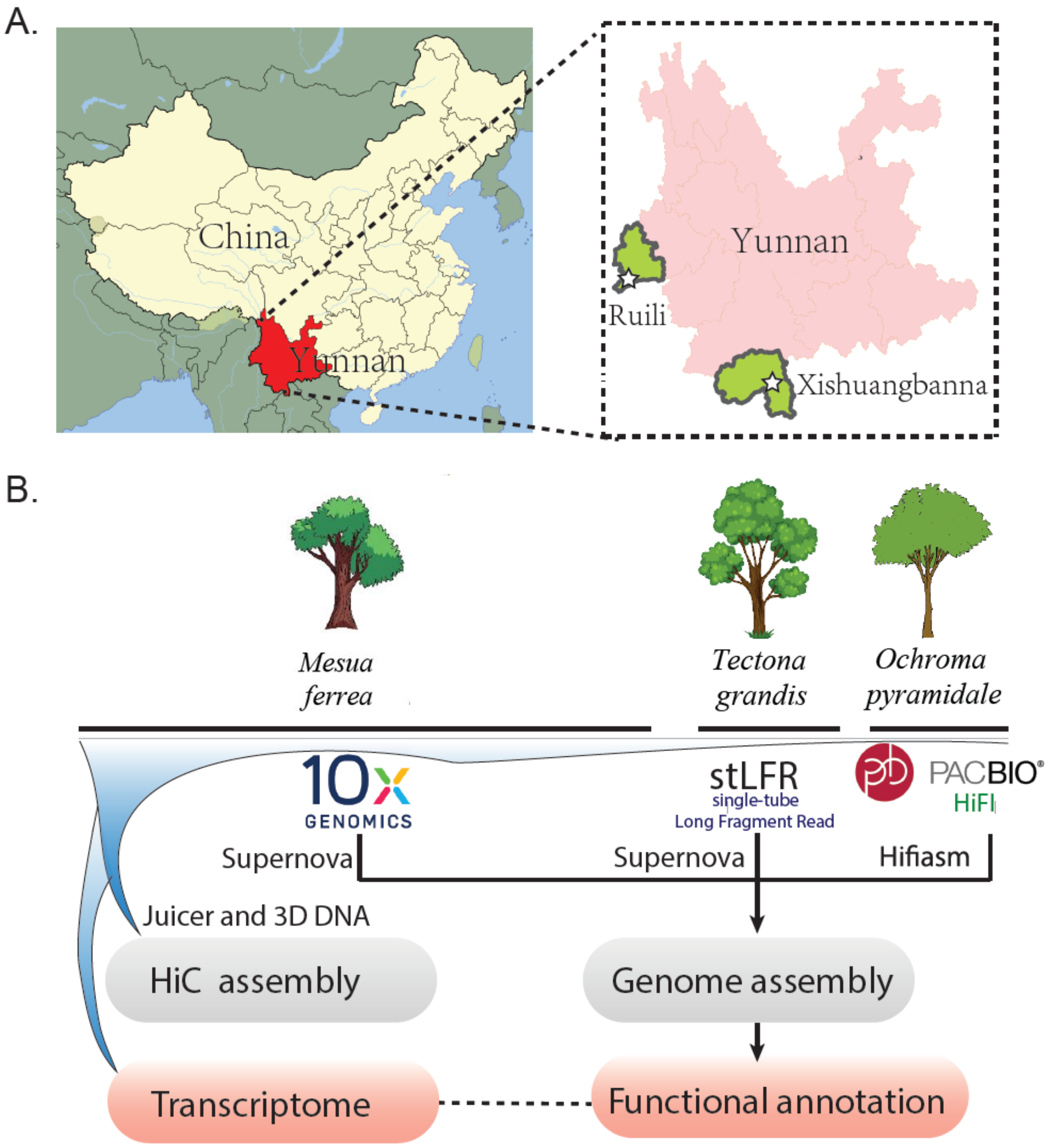

Fig. S4 Sampling sites and genome analysis workflow.

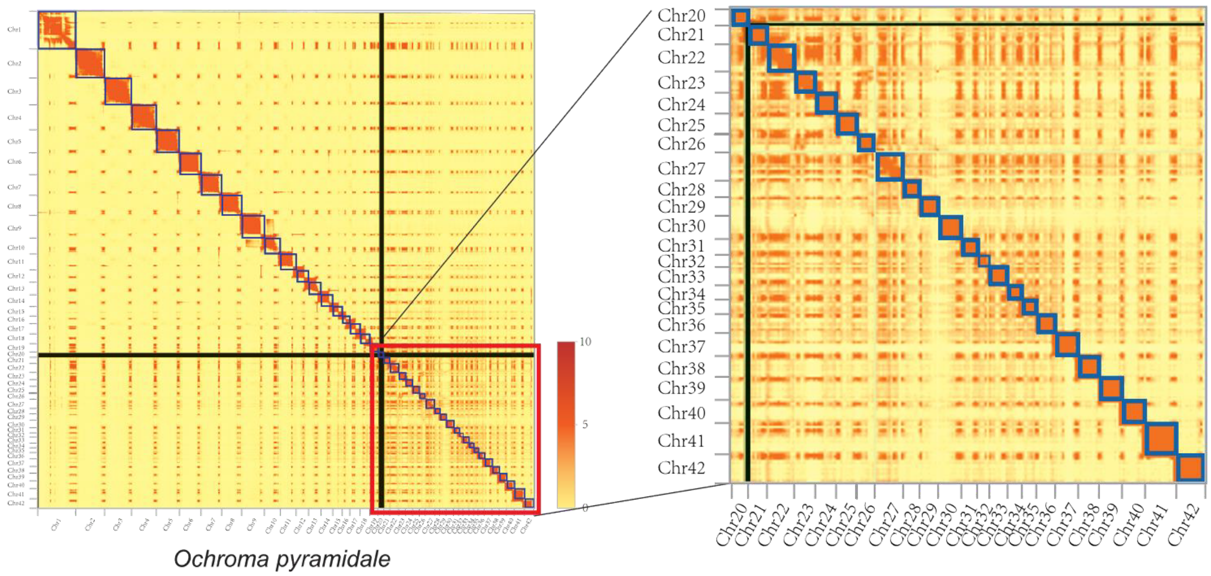

Fig. S5 The Hi-C map of *Ochroma pyramidale*.

The figure shows the genome-wide all-by-all interactions captured by the Hi-C map. The map showcases the detailed structure of individual chromosomes, which are scaffolded and assembled independently. The heat map exhibits a color gradient ranging from light orange to dark orange, representing the varying frequencies of Hi-C interaction links, with lighter colors indicating lower frequencies (0) and darker colors indicating higher frequencies (10).

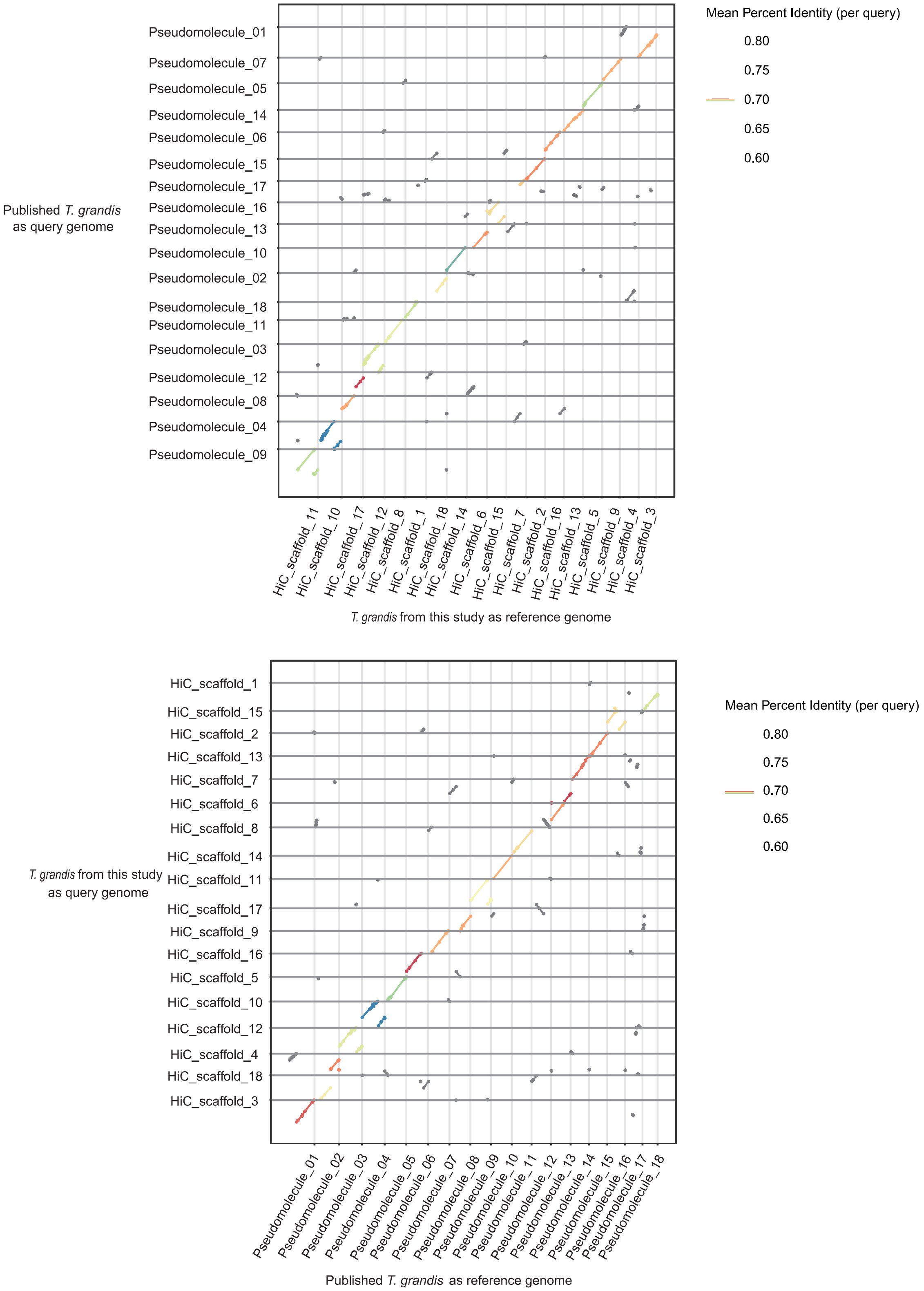

Fig. S6 Sequence alignment dot plots generated with NUCmer (version 4.0.0rc1) comparing the chromosome sequences of *Tectona grandis* from this study to the previously published version.

Forward and reverse aligned sequences are depicted with red and blue lines, respectively. Misplaced scaffolds are indicated by horizontal or vertical shifts in portions of the aligned blocks.
